# Spatial and Temporal Characteristic Analysis and Risk Assessment of Global Highly Pathogenic Avian Influenza H5N8 Subtype

**DOI:** 10.1155/2024/5571668

**Published:** 2024-05-10

**Authors:** Qi An, Yuepeng Li, Zhuo Sun, Xiang Gao, Hongbin Wang

**Affiliations:** ^1^College of Veterinary Medicine, Northeast Agricultural University, Harbin, China; ^2^Key Laboratory of the Provincial Education Department of Heilongjiang for Common Animal Disease Prevention and Treatment, College of Veterinary Medicine, Northeast Agricultural University, Harbin, China

## Abstract

H5N8 HPAI is a highly infectious avian disease that now poses a serious threat and potential risk to poultry farming, wild birds, and public health. In this study, to investigate the seasonality and transmission directionality of global H5N8 HPAI, the spatial and temporal analysis of H5N8 HPAI was conducted using time series decomposition and directional distribution analysis. An ecological niche model was developed for H5N8 HPAI in poultry to identify areas at high risk of H5N8 HPAI in poultry and associated risk factors. The results indicated that three global pandemics of H5N8 HPAI emerged from 2014 to 2022, all showing a southeast–northwest distribution direction. H5N8 HPAI occurred more frequently in winter and less frequently in summer. The southwestern border region and the southeastern region of North America, the southern region of South America, most of Europe, the southern border region and the northern border region of Africa, and the southwestern region and the southeastern region of Asia provide the suitable environment for the occurrence of H5N8 HPAI in poultry. Chicken density, duck density, population density, bio1 (annual mean temperature), and land cover were considered important variables for the occurrence of H5N8 HPAI in poultry. This study can help optimize the use of resources and provide new information for policymakers to carry out prevention and control efforts.

## 1. Introduction

Avian influenza is an acute infectious disease caused by the avian influenza viruses that cause respiratory or systemic infection in avians [1]. Avian influenza viruses are members of the genus *Alphainfluenzavirus* in the family *Orthomyxoviridae* and are classified into different subtypes based on antigenic differences in their surface hemagglutinin (HA) and neuraminidase (NA) [2]. In addition, according to the pathogenicity of the virus to poultry, they can be divided into highly pathogenic avian influenza virus (HPAIV) and low pathogenic avian influenza virus (LPAIV) [3]. Highly pathogenic avian influenza is currently caused by the H5 or H7 hemagglutinin subtype of the virus. Because of its rapid onset, rapid transmission, and high mortality, it has been designated as a notifiable disease by the World Organisation for Animal Health (WOAH) [4]. Over the past few years, outbreaks of H5 subtype highly pathogenic avian influenza have been recurring in wild birds and poultry around the world, and some can even be transmitted to mammals, including humans [5]. The H5N1 highly pathogenic avian influenza virus (A/goose/Guangdong/1/1996) originated in Guangdong Province, China [6]. Over time, the virus has evolved into 10 branches (0–9) and many sub-branches [7]. H5N1 in poultry and wild birds underwent continuous mutation and recombination to produce different subtypes of H5Nx viruses, including H5N2, H5N3, H5N5, H5N6, H5N8, and H5N9 [8, 9].

Avian influenza viruses can be excreted from the feces and respiratory secretions of infected birds and are transmitted between birds by the fecal–oral route and the respiratory route [10–12]. Compared with H5N1 HPAI, H5N8 HPAI was more excreted in wild ducks and could reach the level of direct contact transmission [13]. Intercontinental epidemics of H5N8 HPAI in recent years have been attributed to the long-distance migration of infected wild birds [14], but the specific mechanism of H5N8 HPAIV transmission from wild birds to poultry remains to be investigated. Most outbreaks of H5N8 HPAI in poultry in Europe have occurred on indoor farms, and direct contact between wild birds and poultry seems unlikely. A more reasonable hypothesis is that the HPAI H5N8 virus in the environment is indirectly introduced into poultry farms through humans, vehicles, pollutants, etc. [13].

Since 2010, the 2.3.4 branch of the avian influenza virus has gradually become dominant in the pandemic. In 2010, the H5N8 HPAIV of branch 2.3.4 (A/duck/Jiangsu/k1203/2010) was first isolated from poultry in live poultry markets in Jiangsu, China, but did not cause a large-scale outbreak [15–17]. In the following decade, the H5N8 HPAI caused three large-scale intercontinental epidemics, which affected many countries and regions in Asia, Europe, Africa, and North America [2, 4].

The first intercontinental epidemic began in 2014 with an outbreak of H5N8 HPAI in poultry and wild birds in South Korea, affecting 782 farms in the country over a year [18, 19]. Subsequently, there were varying degrees of H5N8 HPAI outbreaks in Japan, China, Germany, the Netherlands, the United Kingdom, the United States, and Canada [20–25]. In May and June 2016, H5N8 HPAIV was found in dead wild birds in China and Russia, from which the second global H5N8 HPAI outbreak began [26, 27]. The affected countries include dozens of countries and regions in Asia (India, Iran, Saudi Arabia, etc.), Europe (Russia, France, Germany, etc.), and Africa (Uganda, Congo, South Africa, etc.) [28–37]. Compared to the previous outbreak, this one was more widespread, lasted longer, and caused serious economic losses. On December 31, 2019, Poland experienced an outbreak of avian influenza caused by the H5N8 HPAIV of 2.3.4.4b branch, marking the beginning of the third intercontinental epidemic [38]. After that, the H5N8 HPAI epidemic broke out in European countries such as Romania, Slovakia, Czech Republic, Bulgaria, Germany, Hungary, and Israel; Middle East countries such as Iraq, Iran, and Saudi Arabia; and Asian countries such as South Korea, Japan, and China [39–46]. The epidemiological trend of H5N8 HPAI shows that the geographical area affected by it is gradually expanding.

The global spread of H5N8 HPAI has now become a major concern for the international community. Due to poultry death, trade restrictions, and control measures, H5N8 HPAI has caused significant economic losses to the global poultry industry. During the 2016–2017 H5N8 HPAI pandemic in Europe, French authorities adopted strict control measures that resulted in the culling of approximately 5.4 million Anatidae and 1.3 million chickens [47]. Restrictions on international trade in poultry products were also seriously affecting the profitability of the poultry-related industry in France [48]. More than 2.7 million poultry had to be culled in Hungary to control the disease [47]. The 2020 outbreak of H5N8 HPAI on the Eurasian continent led to the deaths or culling of over 20 million poultry in South Korea and Japan [5]. In addition, the continuous mutation of the surface sites of the H5N8 virus has gradually revealed its ability to spread across species. H5N8 has been found to infect ferrets, seals, and humans [49–51]. Although people infected with H5N8 are asymptomatic, the possibility of increased pathogenicity of H5N8 in humans in the future cannot be ruled out. Given the serious threat and potential risk of H5N8 to poultry farming, wild birds, and public health, it should be monitored continuously and vigilantly to prevent a cross-species pandemic of H5N8 HPAI.

Space and time are two important attributes of animal infectious disease events. Spatial and temporal analysis techniques are helpful to explore the epidemic patterns and risk factors of diseases [52]. The analysis of the spatial and temporal distribution characteristics of the historical epidemic can provide a powerful supplement for clinical and molecular biology research [53]. Previous spatial and temporal studies on H5N8 HPAI have been conducted but mostly for localized countries and regions [3, 48, 54, 55]. Local prevalence patterns may not be representative of the whole, and spatial and temporal analysis of H5N8 HPAI in global regions is necessary. In this study, the seasonality and trend of global H5N8 HPAI occurrence were analyzed using the time series decomposition technique. Directional distribution analysis (standard deviation ellipse) was employed to determine the global distribution of H5N8 HPAI and the directionality of transmission. Finally, bioclimatic, geographic landscape, and anthropogenic variables were selected to establish an ecological niche model for H5N8 HPAI in poultry, to identify high-risk areas and associated risk factors for H5N8 HPAI in poultry.

## 2. Materials and Methods

### 2.1. Collection of Epidemic Occurrence Data

Global outbreak data for the H5N8 HPAI in poultry from January 1, 2014, to December 31, 2022, were obtained from the Food and Agriculture Organization of the United Nations (FAO, https://empres-i.apps.fao.org/). The collected information includes the location, time, and specific latitude and longitude coordinates of H5N8 HPAI events. The collected data were cleaned to eliminate records with incomplete and duplicate information, leaving a final total of 6,955 events.

### 2.2. Time Series Decomposition

Time series decomposition allows analysis of the characterization of temporal patterns of disease event occurrence, including trends as well as seasonality [56]. An exploratory analysis of H5N8 HPAI events was first performed using smoothing curves to visually assess approximate trends. The smoothing process was implemented by generalized additive model (GAM). The collected outbreak data were aggregated and organized to generate a time series of H5N8 HPAI events. The seasonal and trend decomposition using loess (STL) method was used to perform the time series decomposition. STL is a decomposition model based on the additive principle with the expression *Y*_[*t*]_=*T*_[*t*]_+*S*_[*t*]_+*R*_[*t*]_, where *Y*_[*t*]_ is the model output at time *t*, *T*_[*t*]_, *S*_[*t*]_, and *R*_[*t*]_ represent the trend component, seasonal component, and residuals at time t, respectively. The time series decomposition was completed in R 4.2.3.

### 2.3. Directional Distribution Analysis

The directional distribution of historical H5N8 HPAI outbreaks was analyzed using the standard deviation ellipse method to determine the direction of its spread. This method can create an ellipsoidal polygon by calculating the standard distance of a set of case points. Based on the long and short axes of the ellipse and the rotation angle of the ellipse, we can determine whether the distribution of H5N8 HPAI outbreak points is directional. According to the global prevalence situation of H5N8 HPAI, the study period was divided into three phases: 2014–2015, 2016−2019, and 2020–2022. Each standard deviation ellipse contains of approximately 68% H5N8 HPAI event points of one phase. Directional distribution analysis is done in the Directional Distribution (standard deviational ellipse) (spatial statistics) function of ArcGIS 10.2 (https://desktop.arcgis.com/en/arcmap/latest/tools/spatial-statistics-toolbox/directional-distribution.htm).

### 2.4. Ecological Niche Modeling

#### 2.4.1. Filtering of Epidemic Occurrence Points

To reduce the impact of spatial autocorrelation on the analysis, the poultry H5N8 HPAI occurrence points were filtered using ENMTools software to ensure that there was only one occurrence point in each grid [57]. Finally, 1,704 poultry H5N8 HPAI occurrence points were retained for subsequent modeling.

#### 2.4.2. Variable Collection and Processing

Previous studies have emphasized three main types of factors associated with the occurrence of avian influenza [55, 58, 59]: (a) anthropogenic variables (e.g., host density and distance to roads and cities), human-mediated activities can have an impact on the spread of avian influenza viruses; (b) bioclimatic variables, the occurrence of avian influenza is associated with ecoclimatic variables (e.g., temperature and precipitation) at different periods; and (c) geographic landscape variables (e.g., land cover and elevation) that represent combinations of the above factors, including the geographic environment in which both human-related activities and wild birds survive. The variables used for ecological niche modeling were shown in Table 1. bio1 (annual mean temperature), bio12 (annual precipitation), wind speed, and elevation were obtained from WorldClim2.1. Land cover data from Esri's Sentinel-2 10 m land use/land cover time series of the world (https://livingatlas.arcgis.com/landcoverexplorer) include nine types of land cover (Table S1). Global population density data was downloaded from LandScan (https://landscan.ornl.gov/). Global density data for chickens and ducks were obtained from the Food and Agriculture Organization of the United Nations livestock systems (https://www.fao.org/livestock-systems/en/). Global road vector data were provided by the Resource and Environment Science and Data Platform (https://www.resdc.cn/Default.aspx). We conducted kernel density analysis on road vector data with a search radius of 10 km and converted it into raster data for backup. The mask extraction and resampling tool were used in ArcGIS 10.2 to make all raster data the same in range and pixel size (all 5 arcminutes, about 10 km). Finally, VIF and Pearson correlation coefficients were tested for all variables in R software to ensure that VIF <5 and the absolute value of Pearson correlation coefficient <0.7 were satisfied between the variables used for modeling, to reduce multicollinearity and correlation. The results of the VIF and Pearson correlation coefficient tests are shown in Table S2 and Figure S1.

#### 2.4.3. Modeling Procedure

In the field of epidemiology, ecological niche models have been widely used to map the distribution of diseases [60]. In this study, MaxEnt 3.4.4 software was employed to predict the global potential distribution of H5N8 HPAI in poultry [61] (https://biodiversityinformatics.amnh.org/open_source/maxent/). The specific model parameters were as follows: the regularization multiplier was one, the features were automatically selected by the model based on the size of the data, the data was split into training data (75%) and test data (25%), and the model output format was logistic. Using Bootstrap as the replicated run type, ran the model 100 times to reduce the error, and took the average of the 100 times results as the final prediction. The area under the receiver operating characteristic curve (AUC) was used to assess the model, with higher values indicating better model performance.

## 3. Results

### 3.1. Temporal Characteristics of H5N8 HPAI Occurrence

**Figure 1:**
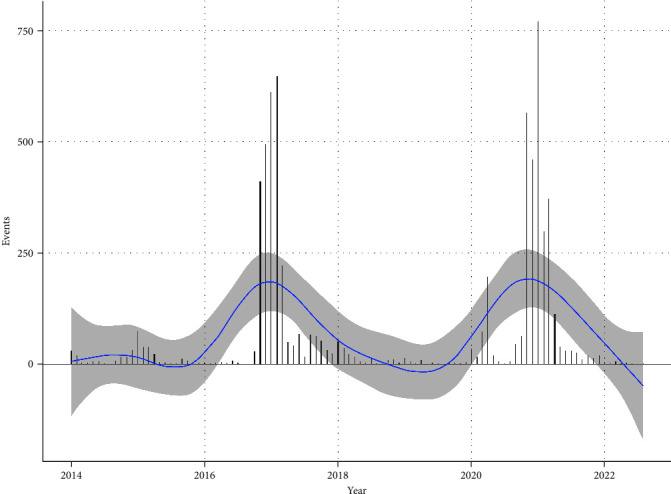
Global H5N8 HPAI events in poultry and wild birds from 2014 to 2022. The blue line depicts GAM-smoothed curve of events, and the gray areas depict a 95% confidence interval.

**Figure 2:**
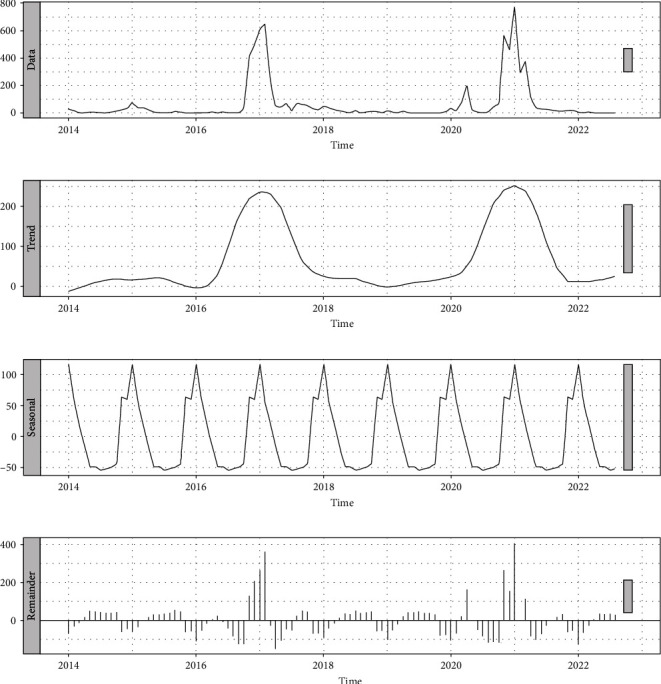
Components of the time series of H5N8 HPAI monthly events for global poultry and wild birds from 2014 to 2022. (a) Time series. (b) Trend component. (c) Seasonal component. (d) Remainder component.

From 2014 to 2022, there were three epidemic peaks of H5N8 HPAI worldwide (Figure 1). The decomposition results of the H5N8 HPAI time series based on STL are shown in Figure 2. The prevalence trend of H5N8 HPAI was evident and largely consistent with the smoothing curve. There were fewer incidents of H5N8 HPAI with a small peak in 2014–2015. And in 2016–2019 and 2020–2022, H5N8 HPAI spread rapidly, showing a pandemic trend. The seasonal cycle of the occurrence of H5N8 HPAI was also apparent, with its spread being the lowest in July and peaked in January. Most H5N8 HPAI outbreaks occurred in the northern hemisphere (1609/1704), suggesting that H5N8 HPAI is favored in winter.

### 3.2. Analysis of the Directional Distribution of H5N8 HPAI

**Figure 3:**
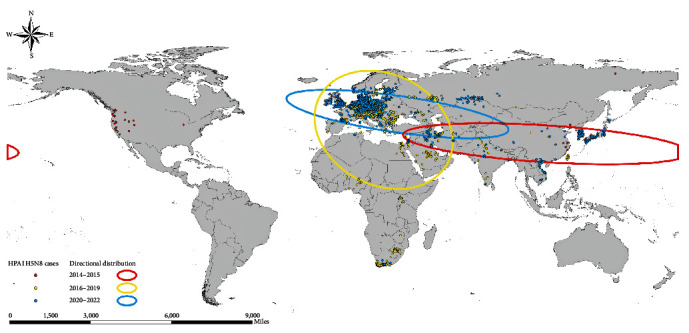
Directional distribution analysis of H5N8 HPAI events for global poultry and wild birds from 2014 to 2022. The points and ellipses represent the H5N8 HPAI events and standard deviation ellipses for the different phases. The red color depicts 2014–2015, the yellow color depicts 2016–2019, and the blue color depicts 2020–2022.

Figure 3 and Table S3 reveal the standard deviation ellipses for each stage of H5N8 HPAI from 2014 to 2022 and the attributed values of the standard deviation ellipses, respectively. The 2014–2015 H5N8 HPAI epidemic was characterized by small regional epidemics in eastern Asia, Europe, and North America, with a large flatness of the ellipse (the ratio of the difference between the long and short semi-axes to the long semi-axis), showing a clear southeast–northwest distribution direction, with the center of the circle located in central China. From 2016 to 2019, H5N8 HPAI was widespread in Asia, Europe, and Africa with a southeast–northwest distribution direction, and the center of the circle is located in Greece. In 2020–2022, H5N8 HPAI mainly showed a pandemic trend in Eurasia, with an overall distribution direction which is southeast to northwest, and the center of the circle is located in the southeast of Moldova.

### 3.3. Model Evaluation and Variable Contributions

**Figure 4:**
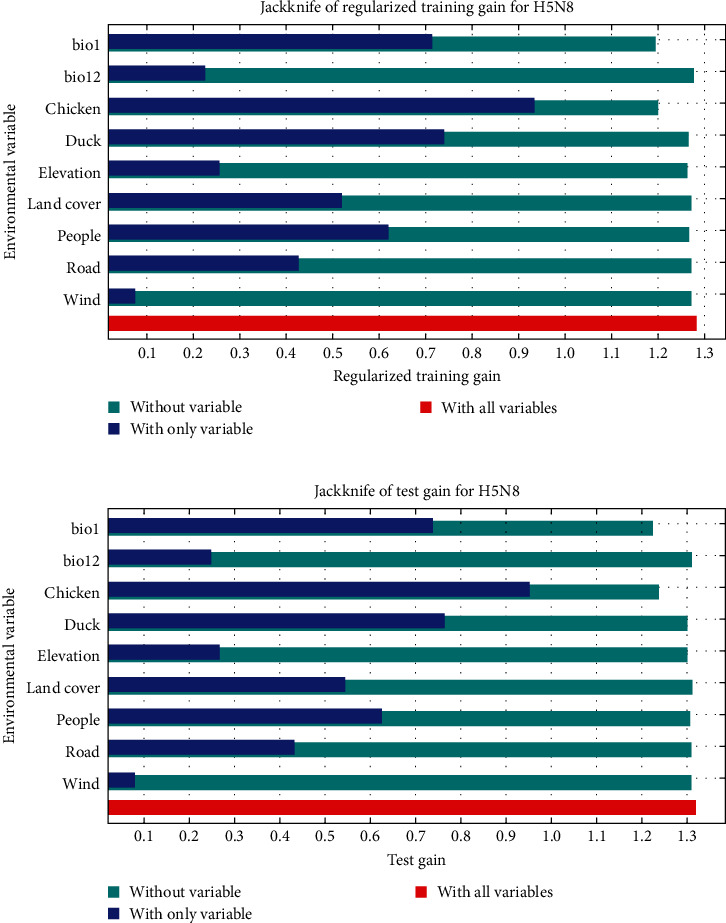
Results of the jackknife test for the variable importance of poultry H5N8 HPAI model. The cyan color depicts the gain of the model constructed with other variables besides this one, the blue color depicts the gain of the model constructed with this variable only, and the red color depicts the gain of the model constructed with all variables. (a) Train gain. (b) Test gain.

The evaluation results of the poultry H5N8 HPAI model are shown in Figure S2. With an average AUC of 0.906 for 100 replicate runs, the model performed well.  Figure 4 shows the results of the jackknife test for the variable importance. Both in terms of training gain and test gain, chicken density, duck density, population density, bio1 (annual mean temperature), and land cover had higher gains when used alone.  Table 2 shows the percentage contributions of variables, with chicken density, bio1 (annual mean temperature), population density, land cover, and duck density being the top five contributing variables. Thus, we identified these five variables as important variables in the poultry H5N8 HPAI model.

### 3.4. Response Curves of Important Variables

**Figure 5:**
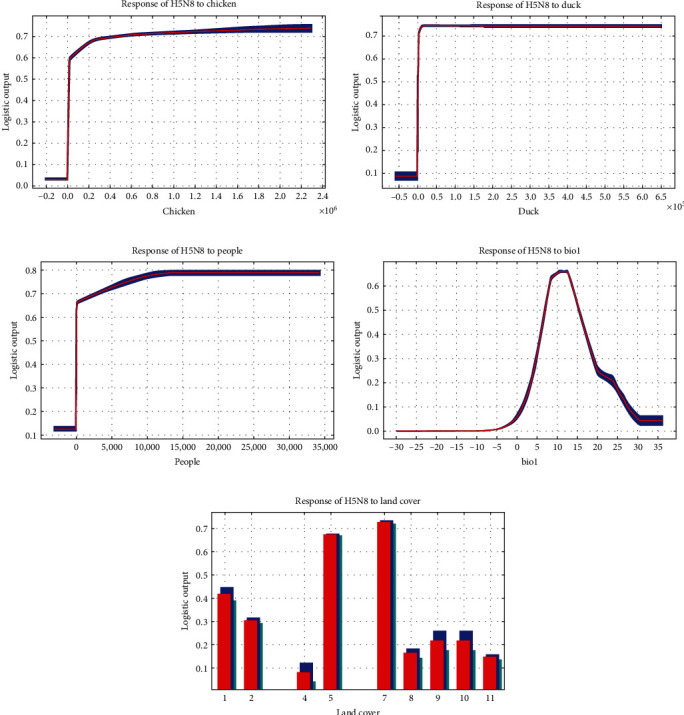
Response curves for important variables of poultry H5N8 HPAI model. The curves show the mean response of the 100 replicate maxent runs (red) and the mean +/- one standard deviation (blue). (a) Chicken. (b) Duck. (c) People. (d) bio1 (annual mean temperature). (e) Land cover (the land cover specific categories with corresponding numeric codes are shown in Table S1).

Figure 5 illustrates the response curves of important variables in the poultry H5N8 HPAI model. The curves for chicken density, duck density, and population density showed similar trends, with the probability of H5N8 HPAI increasing with the horizontal coordinate and then remaining flat. For bio1 (annual mean temperature), the occurrence probability of H5N8 HPAI showed a trend of first increasing and then decreasing with the increase, reaching a peak value at about 8.5–12.5°C. According to the response curve of land cover, it is known that crops and built areas are suitable for the occurrence of H5N8 HPAI.

### 3.5. H5N8 HPAI Occurrence Suitability Map

**Figure 6:**
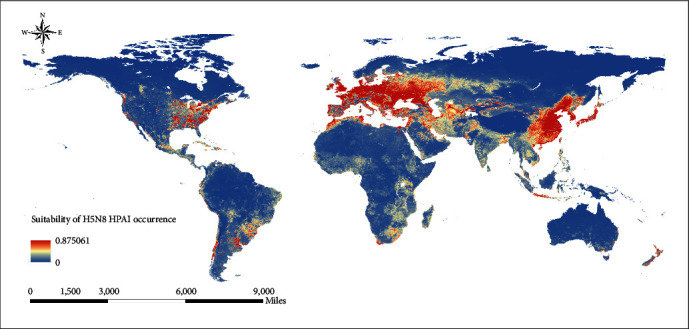
Suitability map for the H5N8 HPAI occurrence of poultry. The warmer colors depict areas of high suitability, while the cooler colors depict areas of low suitability.

The ecological niche model accurately identified the environmental conditions suitable for the occurrence of H5N8 HPAI in poultry. Predictions indicate that the southwestern border region and the southeastern region of North America, the southern region of South America, most of Europe, the southern border region and the northern border region of Africa, and the southwestern region and the southeastern region of Asia are at high risk for the occurrence of H5N8 HPAI in poultry (Figure 6).

## 4. Discussion

H5N8 HPAI is a notifiable infectious disease, and its successive outbreaks around the world have caused serious damage to the poultry industry, the health of wild birds, farmers' livelihoods, and international trade. The continuous mutation and evolution of H5N8 HPAIV, as well as the storage and long-distance transmission of H5N8 HPAIV by wild birds [2, 14], pose difficulties for the prevention and control of H5N8 HPAI. To address this current situation, this study combined spatial and temporal analysis techniques to analyze the spatial and temporal epidemiological characteristics of global H5N8 HPAI and predicted suitable areas for the occurrence of H5N8 HPAI in poultry, intending to provide some reference for policymakers to formulate corresponding measures.

The results of time series decomposition for global H5N8 HPAI show that the prevalence of H5N8 HPAI has a clear trend and seasonality. H5N8 HPAI showed a small peak in 2014–2015 and a pandemic trend in 2016–2019 and 2020–2022, which largely coincided with the three intercontinental epidemics described in previous studies [2, 4]. Beginning in 2020, H5N1 HPAI outbreaks began to grow significantly each year. H5N1 HPAI outbreaks accounted for more than 90% of the global outbreaks of highly pathogenic avian influenza in the last 2 years (FAO, https://empres-i.apps.fao.org/). The dominant subtype of highly pathogenic avian influenza globally has shifted from H5N8 HPAI to H5N1 HPAI [62]. Although the global occurrence of H5N8 HPAI is currently at a low point, based on the trend chart, it is speculated that a new epidemic peak of H5N8 HPAI is likely to occur in the coming years. H5N8 HPAI shows similar seasonal characteristics to other H5 subtypes of avian influenza, with outbreaks occurring mostly in the cold winter and early spring periods, and relatively few outbreaks in the summer and autumn [63]. Ottaviani's study showed that low temperatures affected wild bird migration patterns and determined wild bird distribution, which in turn affected the spread of highly pathogenic avian influenza [64]. Cold weather can increase the gathering of wild birds [64], potentially allowing the H5N8 HPAIV to persist in the environment. Napp's study also indicated that cold temperatures from October 2016 to January 2017 may have been a driver of H5N8 expansion in southern and western Europe in 2016–2017 [47].

The analysis of the directional distribution of historical H5N8 HPAI outbreaks revealed that 2014–2015, 2016−2019, and 2020–2022 all showed a southeast–northwest distribution direction, with only slight differences in the flatness and rotation angle of the ellipse. All three phases are long-range intercontinental epidemics, and the shifting trend in the center of the standard deviation ellipse reflects the overall shifting direction of the H5N8 HPAI epidemic in each phase. H5N8 HPAI was first prevalent in small regions of eastern Asia, Europe, and North America; then spread widely in Asia, Europe, and Africa; and finally broke out in Eurasia. The distribution direction of H5N8 HPAI may be closely related to the migratory path of wild birds. Infected wild birds flew from Korea to their northern breeding grounds in the spring of 2014 and migrated to wintering sites in Europe and North America in autumn, which is the most plausible explanation for the large-scale geographic spread of H5N8 HPAI in 2014 [14]. Zhang's epoch discrete phylogeographic model also demonstrated that wild bird migration networks were positively associated with H5N8 HPAIV transmission since early 2020 and were the key driver of the bidirectional movement of H5N8 HPAIV between Europe and Asia [65].

To determine the high-risk areas and risk factors for H5N8 HPAI in poultry, we used bioclimate, geographical landscape, and anthropogenic variables to establish a maximum entropy model for H5N8 HPAI in poultry. Based on the results of the jackknife test of variable importance and the percentage contribution, chicken density, duck density, population density, bio1 (annual mean temperature), and land cover had significant effects on H5N8 HPAI in poultry. From the response curve, the occurrence probability of H5N8 HPAI gradually increased with the increase of chicken density, duck density, and population density, and the curve remained stable after reaching a specific value, with an overall roughly positive correlation trend. The number of poultry flocks is a risk factor for highly pathogenic avian influenza [66], and an increase in poultry population contributes to the spread of highly pathogenic avian influenza [67]. Kim's study also found similar results, with “farm with ≥ seven poultry flocks” and “a flock size > 2000” being risk factors for H5N8 HPAI outbreaks in duck farms in South Korea [68]. A possible explanation is that the higher the density of birds, the higher the frequency of contact with birds for persons carrying H5N8 HPAIV. And farms with large flocks of chickens and ducks may be difficult to disinfect thoroughly. The effect of population density on H5N8 HPAI is similar to that on H5N1 HPAI. Gilbert and Pfeiffer [58] reviewed spatial analysis studies of H5N1 HPAI, in which H5N1 HPAI occurrence was positively correlated with population density in many countries and regions. Densely populated areas may represent more intensive live poultry trade and associated agricultural activities, increasing the probability of detection and spread of avian influenza outbreaks [58]. The response curve of bio1 (annual mean temperature) shows that the occurrence of H5N8 HPAI is preferred at lower mean annual temperatures (about 5−19°C). However, the annual mean temperature is an arithmetic mean, and the specific temperature suitable for the survival of H5N8 HPAIV needs to be further studied. A study has shown that the highly pathogenic avian influenza viruses can survive longer at lower temperatures [69]. Combined with the seasonal characteristic that H5N8 HPAI is more frequent in winter, it seems that places with lower mean annual temperatures may have colder winters, thus providing suitable conditions for the spread of H5N8 HPAI. The response curve of land cover illustrates the types of land cover suitable for the occurrence of H5N8 HPAI, including crops and built areas. Crops represent crops humans grow, such as rice, wheat, and soybeans. The study revealed that rice fields on the one hand represent the distribution of free-grazing ducks and on the other hand provide habitat for wild birds [70, 71]. Crops may increase the chance of direct contact between free-grazing poultry and wild birds, which also increases the risk of H5N8 HPAI transmission. Built areas refer to human-made structures, such as dense villages, towns, and cities. Built areas were similar to the effects caused by population density on H5N8 HPAI. Built areas represent the distribution and range of human activity and often imply frequent poultry-related social activities.

The significance of ecological niche modeling in infectious diseases lies in its ability to explore the relationship between disease occurrence and climate and environmental variables and to map this relationship to new regions, thereby predicting the distribution of diseases. The risk map of H5N8 HPAI in poultry shows that the high-risk areas for the occurrence of H5N8 HPAI in poultry are mainly distributed in the midlatitude areas (30°−60°). These areas are located in temperate zones, mostly characterized by cold winters and hot summers, which are compatible with the seasonal characteristics of the H5N8 HPAI outbreak. Notably, although no H5N8 HPAI has ever occurred in South America, the suitability map reveals a high risk for the occurrence of H5N8 HPAI in the southern region of South America. These areas possess climate and environmental conditions suitable for the occurrence of H5N8 HPAI. Once introduced, there is a high likelihood of triggering a series of outbreaks. For high-risk areas, first of all, poultry immunization should be carried out on schedule, and strict biosecurity measures should be implemented. Secondly, wild bird monitoring should be strengthened to keep poultry away from wild bird contact. In addition, poultry trade activities should be strictly monitored, and virus sampling and testing should be conducted regularly to detect the H5N8 HPAI outbreak on time and prevent its rapid spread.

There are some limitations to our study. Due to the lack of relevant data, wild bird migration data were not used in the establishment of the poultry H5N8 HPAI niche model. The migration of wild birds may have played a crucial role in the intercontinental prevalence of H5N8 HPAI in recent years [14]. Although we selected variables that may be relevant to wild bird ecology (wind speed, elevation, and land cover), they can only reflect their habits to a certain extent. On the other hand, the historical outbreak data of H5N8 HPAI in our study, all from official reports, may differ from the actual outbreak data. If there is underreporting in the official data, then some of the findings in this study may be biased.

## 5. Conclusion

From 2014 to 2022, there were three intercontinental epidemics of H5N8 HPAI in the world, all showing a southeast-to-northwest distribution direction. The seasonal cycle of H5N8 HPAI occurrence was obvious, mostly in winter and less in summer. The results of the ecological niche model suggested that the southwestern border region and the southeastern region of North America, the southern region of South America, most of Europe, the southern border region and the northern border region of Africa, and the southwestern region and the southeastern region of Asia are at high risk for the occurrence of H5N8 HPAI in poultry. Chicken density, duck density, population density, bio1 (annual mean temperature), and land cover are key factors influencing the occurrence of H5N8 HPAI in poultry. This study has strategic significance for the prevention and control of H5N8 HPAI.

## Figures and Tables

**Table 1:** Variables used in the model.

Variable classification	Variable code	Variable name	Source
Bioclimatic	bio1	Annual mean temperature	WorldClim version 2.1
	bio12	Annual precipitation	WorldClim version 2.1
	Wind	Wind speed	WorldClim version 2.1

Geographic landscape	Elevation	Elevation	WorldClim version 2.1
	Land cover	Land cover	Esri

Anthropogenic	People	People density	LandScan
	Chicken	Chicken density	FAO livestock system
	Duck	Duck density	FAO livestock system
	Road	Road density	Resource and Environment Science and Data Platform

**Table 2:** Percent contribution of variables in poultry H5N8 HPAI model.

Variable	Percent contribution (%)
Chicken	60
bio1	9.6
People	8.1
Land cover	5.7
Duck	5.6
Road	5.5
Wind	3.3
Elevation	2
bio12	0.2

## Data Availability

The datasets generated and/or analyzed during the current study are available from the corresponding author upon reasonable request.

## Supplementary Materials

Supplementary Materials Figure S1: heatmap of Pearson correlation coefficient between variables. The cooler colors depict a higher positive correlation while warmer colors depict a higher negative correlation. Figure S2: evaluation results of poultry H5N8 HPAI model based on the area under the receiver operating characteristic curve. The curves show the mean ROC of the 100 replicate maxent runs (red) and the mean +/− one standard deviation (blue). The black line indicates random prediction. Table S1: land cover specific categories with corresponding numeric codes. Table S2: results of VIF test for variables. Table S3: attributed values for the global H5N8 HPAI evevts standard deviation ellipse, 2014–2022.
