# Supplementary material for: Spatial and Temporal Characteristic Analysis and Risk Assessment of Global Highly Pathogenic Avian Influenza H5N8 Subtype
Source: Transbound Emerg Dis. 2024 May 10;2024:5571668. doi: 10.1155/2024/5571668 (PMC12016704; doi:10.1155/2024/5571668)
Supplement: Supplementary Materials — Figure S1: heatmap of Pearson correlation coefficient between variables. The cooler colors depict a higher positive correlation while warmer colors depict a higher negative correlation. Figure S2: evaluation results of poultry H5N8 HPAI model based on the area under the receiver operating characteristic curve. The curves show the mean ROC of the 100 replicate maxent runs (red) and the mean +/− one standard deviation (blue). The black line indicates random prediction. Table S1: land cover specific categories with corresponding numeric codes. Table S2: results of VIF test for variables. Table S3: attributed values for the global H5N8 HPAI evevts standard deviation ellipse, 2014–2022. [file 5571668.f1.docx]

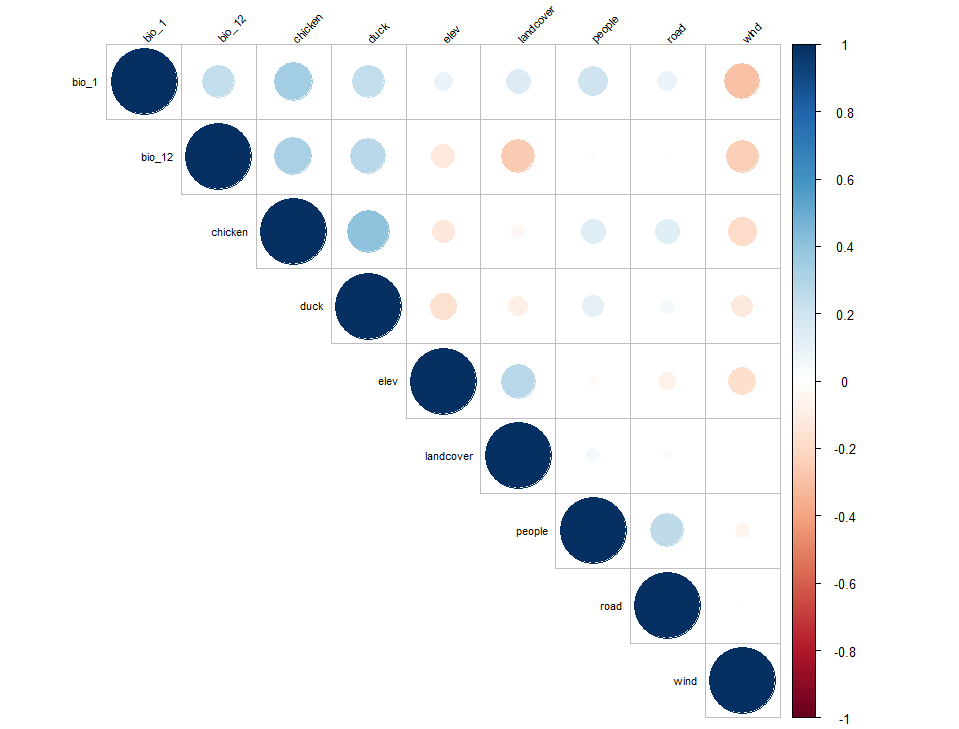
Figure S1. Heatmap of Pearson correlation coefficient between variables. The cooler colors depict a higher positive correlation while warmer colors depict a higher negative correlation.


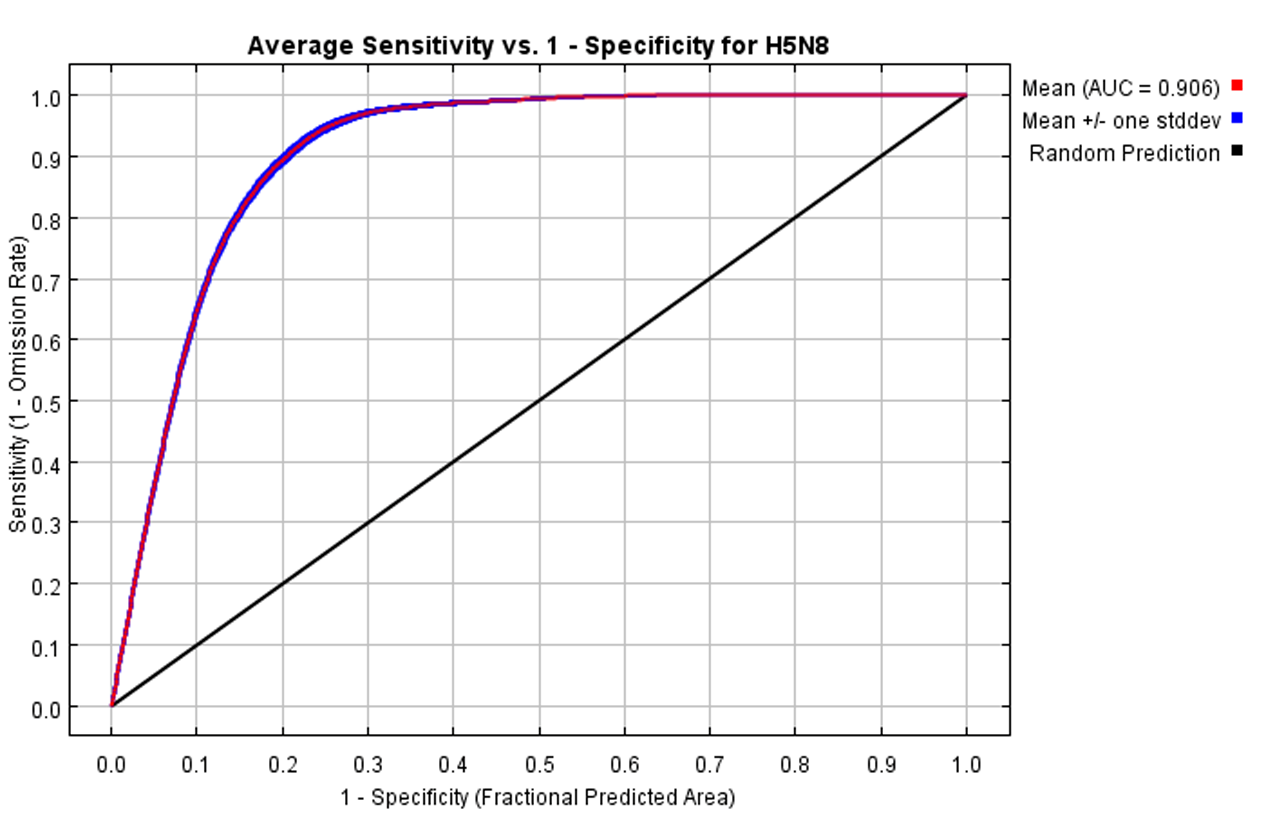


Figure S2. Evaluation results of poultry H5N8 HPAI model based on the area under the receiver operating characteristic curve. The curves show the mean ROC of the 100 replicate maxent runs (red) and the mean +/- one standard deviation (blue). The black line indicates random prediction.

Table S1. Land cover specific categories with corresponding numeric codes

| Code | Categories name |
| --- | --- |
| 1 | Water |
| 2 | Trees |
| 4 | Flooded vegetation |
| 5 | Crops |
| 7 | Built area |
| 8 | Bare groud |
| 9 | Snow or ice |
| 10 | Clouds |
| 11 | Rangeland |

Table S2. Results of VIF test for variables

| Variable | VIF value |
| --- | --- |
| bio_1 | 1.33 |
| bio_12 | 1.32 |
| chicken | 1.38 |
| duck | 1.27 |
| elev | 1.18 |
| landcover | 1.18 |
| people | 1.12 |
| road | 1.09 |
| wind | 1.18 |

Table S3. Attributed values for the global H5N8 HPAI evevts standard deviation ellipse, 2014-2022

| Phase | Center coordinates | XStdDist | YStdDist | Shape Area | Rotation |
| --- | --- | --- | --- | --- | --- |
| Phase 1 2014-2015 | (109.1457938, 30.94431069) | 77.05 | 9.87 | 2386.01 | 93.60 |
| Phase 2  2016-2019 | (21.92812295, 38.41536524) | 38.68 | 29.69 | 3608.25 | 116.23 |
| Phase 3  2020-2022 | (29.34956126, 46.85929157) | 60.24 | 9.79 | 1852.37 | 98.18 |
